# Neuroanatomical correlates of cognitive functioning in prodromal Huntington disease

**DOI:** 10.1002/brb3.185

**Published:** 2013-11-13

**Authors:** Deborah L Harrington, Dawei Liu, Megan M Smith, James A Mills, Jeffrey D Long, Elizabeth H Aylward, Jane S Paulsen

**Affiliations:** 1 Department of Radiology, University of CaliforniaSan Diego, California; 2 Research Service, VA San Diego Healthcare SystemSan Diego, California; 3 Department of Psychiatry, University of Iowa Carver College of MedicineIowa City, Iowa; 4 Seattle Children's Research InstituteSeattle, Washington; 5 Department of Neurology, University of Iowa Carver College of MedicineIowa City, Iowa

**Keywords:** Cognition, magnetic resonance imaging, prodromal Huntington disease

## Abstract

**Introduction:**

The brain mechanisms of cognitive impairment in prodromal Huntington disease (prHD) are not well understood. Although striatal atrophy correlates with some cognitive abilities, few studies of prHD have investigated whether cortical gray matter morphometry correlates in a regionally specific manner with functioning in different cognitive domains. This knowledge would inform the selection of cognitive measures for clinical trials that would be most sensitive to the target of a treatment intervention.

**Method:**

In this study, random forest analysis was used to identify neuroanatomical correlates of functioning in five cognitive domains including attention and information processing speed, working memory, verbal learning and memory, negative emotion recognition, and temporal processing. Participants included 325 prHD individuals with varying levels of disease progression and 119 gene-negative controls with a family history of HD. In intermediate analyses, we identified brain regions that showed significant differences between the prHD and the control groups in cortical thickness and striatal volume. Brain morphometry in these regions was then correlated with cognitive functioning in each of the domains in the prHD group using random forest methods. We hypothesized that different regional patterns of brain morphometry would be associated with performances in distinct cognitive domains.

**Results:**

The results showed that performances in different cognitive domains that are vulnerable to decline in prHD were correlated with regionally specific patterns of cortical and striatal morphometry. Putamen and/or caudate volumes were top-ranked correlates of performance across all cognitive domains, as was cortical thickness in regions related to the processing demands of each domain.

**Conclusions:**

The results underscore the importance of identifying structural magnetic resonance imaging (sMRI) markers of functioning in different cognitive domains, as their relative sensitivity depends on the extent to which processing is called upon by different brain networks. The findings have implications for identifying neuroimaging and cognitive outcome measures for use in clinical trials.

## Introduction

A formal diagnosis of Huntington disease (HD) is made on the basis of unequivocal motor signs. However, subtle motor, psychiatric, and cognitive symptoms are detected years before a motor diagnosis in the prodromal phase (prHD) (Paulsen et al. 2008). Cognitive decline in prHD is of keen interest due to its correlation with genetic markers of disease progression (Tabrizi et al. 2009; Bechtel et al. 2010; Duff et al. 2010; Rowe et al. 2010; Stout et al. 2011) and time to diagnosis (Harrington et al. 2012). With the development of treatments that might prevent or slow the progression of the disease, it is vital to identify cognitive variables for clinical trials that are strongly associated with neurodegeneration and its progression, and that would be sensitive for evaluating treatment effects. In this regard, it is important to identify the brain centers that govern core cognitive functions, as the suitability of a cognitive measure will likely depend on the target of a treatment intervention.

The brain mechanisms of cognitive impairment in prHD are not well understood partly due to the dearth of structural imaging investigations into neurocognitive relationships. Despite progressive changes in the striatum and in cortical gray and white matter that begin decades before a manifest diagnosis (Nopoulos et al. 2010; Paulsen et al. 2010; Aylward et al. 2011; Tabrizi et al. 2011, 2012), much less is known about how they relate to functioning in different cognitive domains. Several studies of prHD have reported that striatal volume (Campodonico et al. 1998; Jurgens et al. 2008; Paulsen et al. 2010; Papp et al. 2013; Wolf et al. 2013) and/or white matter volume (Paulsen et al. 2010; Papp et al. 2013) correlate with measures of executive functioning including the Symbol Digits Modality Test (SDMT), Stroop Interference, Verbal Fluency, the Trial Making Test Part B, and the Towers Task. Yet few studies of prHD have investigated whether cortical gray matter morphometry correlates in meaningful ways with functioning in different cognitive domains. In an early study of 15 prHD individuals that used whole-brain voxel-based morphometry (VBM) (Rosas et al. 2005), linear regression analyses revealed that Verbal Fluency, Stroop Interference, and SDMT performances correlated with cortical thinning in some spatially different regions, suggesting that structural changes were functionally meaningful. Yet a recent study of 20 prHD individuals found no statistically significant relationships between cortical morphometry and cognitive functioning on tests of alertness, divided attention, verbal and spatial working memory, inhibition, or executive dysfunction (Wisconsin Card Sorting Test, WCST), irrespective of the structural magnetic resonance imaging (sMRI) method employed (i.e., VBM and cortical surface modeling) (Wolf et al. 2013). These discrepant findings may relate to the small sample sizes, which is problematic given the heterogeneity of symptoms and disease progression in prHD. Other studies of large combined samples of prHD and manifest HD have revealed relationships between cortical thinning and cognition (e.g., timing, visuomotor integration, emotion recognition) (Bechtel et al. 2010; Say et al. 2011; Scahill et al. 2013). However, the results do not address the neurocognitive relationships in the premanifest period, wherein structural changes in the brain may exhibit more regionally specific relationships with different cognitive functions rather than potentially relate more to global neurodegeneration.

This study of prHD builds upon past research by identifying corticostriatal correlates of functioning in five cognitive domains including attention and information processing speed, working memory, verbal learning and memory, negative emotion recognition, and timing. In intermediate analyses, we first identified regions that showed significant cortical thinning and striatal volume loss in a large sample of prHD individuals relative to a gene-negative control group. These regions were then used as predictors of performance on each cognitive measure in the prHD group. As brain regions interact with each other to fulfill a cognitive function, we hypothesized that performance in each of the domains would be correlated with different regional patterns of corticostriatal morphometry. The random forest method was used to test the hypothesis, as it is well suited for modeling these complicated relationships.

## Material and Methods

### Subjects

Study participants included 325 prHD individuals and 119 gene-negative controls with a family history of HD. Data for the study were collected at 31 sites in the United States, Canada, Australia, Germany, Spain, and the United Kingdom from 2002 to 2008 from individuals enrolled in PREDICT-HD (Paulsen et al. 2006, 2008). Consent was obtained according to the Declaration of Helsinki. The protocol was approved by the institutional review boards at the University of Iowa and each participating site.

Participants were 18 years of age or older, had a family history of HD, and completed independent genetic testing for the HD CAG expansion prior to entry into PREDICT-HD. Confirmatory DNA testing was conducted on blood drawn at the baseline PREDICT visit using a polymerase chain reaction method to determine CAG-repeat length (Warner et al. 1993). PrHD participants had the expansion (≥38 CAG repeats) and gene-negative controls did not (<36 CAG repeats) (1). A certified examiner performed the Unified Huntington's Disease Rating Scale (UHDRS) motor examination on all participants. The UHDRS motor scale contains 31 items that assess chorea, bradykinesia, rigidity, dystonia, and oculomotor function. Ratings for each item range from 0 (normal) to 4 (motor abnormalities, impairment) and are summed for a total motor score (Table 1). Examiners also rated their level of confidence that observed motor signs were an unequivocal manifestation of HD. Individuals with a diagnostic confidence rating of 4 (≥99% confidence that motor symptoms were unequivocal signs of HD) at the time of testing were excluded. Individuals were excluded from participation in PREDICT-HD if they evidenced unstable medical or psychiatric conditions, reported substance abuse within the past year, had a history of learning disability or intellectual disability requiring special education classes, a history of other central nervous system disease (e.g., seizures, traumatic brain injury), or if they had a pacemaker or metallic implants. Individuals were also excluded from participation if they had used prescription antipsychotic medications within the past 6 months or if they used phenothiazine-derivative antiemetic medications more than three times per month, but no other prescription or over-the-counter medications or natural remedies were restricted. All participants underwent comprehensive baseline evaluations including blood draw, neurological/motor examination, cognitive assessment, psychiatric and psychological questionnaires, and brain MRI.

**Table 1:** Characteristics of study participants

	Controls (*n* = 119)	prHD (*n* = 325)	*P*-value
% Women^1^	65.5%	64.9%	0.90
Age (years)^2^	42.4 (11.4)	40.7 (10.2)	0.14
Education (years)^3^	14.7 (2.8)	14.3 (2.7)	0.12
UHDRS motor score^4^	2.7 (3.4)	5.2 (5.5)	0.0003
CAG-repeat length	20.2 (3.3)	42.3 (2.3)	

Means (standard deviations) are reported for all variables except gender. prHD, prodromal Huntington disease; UHDRS, Unified Huntington's Disease Rating Scale.

1 Chi-square test of group differences.

2 Two-sided *t*-test of group differences.

3 Two-sided Wilcoxon test of group differences.

4 Two-sided Kolmogrov–Smirnov test of group differences.

Table 1 shows that age, years of education, and gender were well balanced between the control and the prHD groups. As expected, the prHD group exhibited more symptoms on the UHDRS motor scale than the control group. In the prHD group, the ranges for age (20.1–74.9 years) and CAG-repeat length (38–50) indicate a wide variation in baseline progression levels (Zhang et al. 2011).

### Cognitive tests

PREDICT-HD participants completed a battery of neuropsychological tests and computerized cognitive tasks once a year. From this battery five tests were selected that represent different cognitive domains including attention and information speed, verbal working memory, verbal learning and memory, negative emotion processing, and temporal processing. These domains of cognitive functioning are known to decline in prHD (Rowe et al. 2010; Stout et al. 2011; Harrington et al. 2012). *Attention and processing speed* was measured by the SDMT (total correct in 90 sec) (Smith 1982). *Verbal working memory* was measured by the Wechsler Adult Intelligence Scale-III Letter-Number Sequencing (total correct) (Wechsler 1997). *Verbal learning and memory* was measured by the Hopkins Verbal Learning Test-Revised (HVLT-R) (immediate recall, total number correct) (Brandt and Benedict 2001). *Negative Emotion processing* was measured using a computerized emotion recognition task where the participant viewed photographs of faces expressing one of six emotions (fear, disgust, happiness, sadness, surprise, anger) or a neural expression, and then matched the facial expression with a verbal description (Johnson et al. 2007). The number correct for the negative emotions was the dependent measure as it best discriminates prHD from control participants (Stout et al. 2011). *Temporal processing* was assessed by the paced timing task (Rowe et al. 2010), wherein the participant starts out by tapping in synchrony with a 550 msec isochronous tone and then continues tapping without the tone at the same pace (continuation phase). The measure of timing precision is the reciprocal of the within-subject intertap interval standard deviation during the continuation phase.

This study reports cross-sectional data from a sample of participants whose cognitive testing coincided with their first PREDICT-HD brain MRI, which was conducted at either the first visit or the third visit. Due to the potential effects of practice on task performance for individuals at their third visit, we tested for the effects of the number of visits and group (control vs. prHD) on performance using an analysis of covariance (ANCOVA) model, covarying age, gender, and education. Performance on all cognitive measures did not differ between individuals who had taken the tests once or three times (*P *> 0.10), nor did the number of visits interact with group (*P* > 0.42). Despite these negative results, number of visits was still included as a covariate in the remaining analyses to adjust for its potential minor influences on the cognitive measures.

### MRI acquisition and preprocessing

All scans were obtained using a standard multimodal protocol that included an axial 3D volumetric spoiled-gradient echo series (∼1 × 1 × 1.5 mm voxels) and a dual echo proton density/T2 (∼1 × 1 × 3 mm voxels) series. Thirty sites used General Electric 1.5 Tesla scanners, and two sites used Siemens 1.5 Tesla scanners. Each multimodal scan series was processed through BRAINS (Brain Research: Analysis of Image, Networks, and Systems) AutoWorkup (Pierson et al. 2011), a standardized morphometric processing pipeline that corrected for common multisite data differences (Magnotta et al. 2002). Outputs from the processing pipeline included basal ganglia volumes (caudate, putamen), a brain mask used for computing the intracranial volume (ICV), and a T1-weighted image, which was used in FreeSurfer for cortical thickness processing (Fischl et al. 2002). FreeSurfer estimates of cortical thickness demonstrate very good test–retest reliability across scanners and sites (Han et al. 2006; Dickerson et al. 2008; Jovicich et al. 2009; Reuter et al. 2012). The brain mask was derived from all three image intensity modes to obtain robust estimates of ICV, which include tissue and surface cerebrospinal fluid (CSF) that extends to the border of dura mater. To account for individual differences in head size, basal ganglia volumes were divided by ICV. The T1-weighted image was created with isotropic (1.0 mm^3^) voxels. T1 images were normalized so that the tissue intensities across the spatial domain of a single image and scans from different sites were placed in a consistent intensity range. Spatial intensity inhomogeneities were removed by applying a parametric correction (Styner et al. 2000) that used estimates of the tissue intensities based on tissue classes from the multimodal tissue classification (Harris et al. 1999). Each scan's intensity range was placed on a consistent scale by linearly scaling to maximize the dynamic range inside the brain region. A reoriented, inhomogeneity, and intensity-corrected T1 scan for each subject was then clipped to the brain mask to be used as input for cortical parcellation.

Cortical reconstruction was performed using the FreeSurfer image analysis suite (http://surfer.nmr.mgh.harvard.edu), which is an automated tissue classification and segmentation software that exhibits good test–retest reliability across scanner manufactures and field strengths (Han et al. 2006). Each subject's MRI was initially analyzed in original space using the following analysis pipeline. Processing included removal of nonbrain tissue by a hybrid watershed/surface deformation procedure, subcortical structures were segmented (Fischl et al. 2002), and further intensity normalization was conducted. This was followed by white matter segmentation, tessellation of the gray–white matter boundary, and automated topology correction (Fischl et al. 2001). Then surface deformation following intensity gradients optimally placed the gray/white and gray/cerebrospinal fluid borders at the location where the greatest shift in intensity defines the transition to the other tissue class (Fischl et al. 2001). Once the cortical models were complete, deformable procedures performed additional data processing and analysis, including parcellation of the cerebral cortex into 34 conventional gyral-and sulcal-based neuroanatomical regions in each hemisphere (Desikan et al. 2006). This parcellation method demonstrates diagnostic sensitivity in other diseases (Desikan et al. 2009). Intensity and continuity information from the segmentation and deformation procedures produced representations of cortical thickness, which were calculated as the closest distance from the gray–white matter boundary to the gray–CSF boundary at each vertex on the tessellated surface (Fischl and Dale 2000). Cortical thickness was used in this study as it accounts for most volumetric changes in prHD (Nopoulos et al. 2010) and is influenced by genetic factors (Winkler et al. 2010).

### Statistical analyses

We employed the random forest method (Breiman 2001) to identify the relationships between brain morphometric measures and cognition for several reasons. First, there are a large number of variables (brain regions) and many of them are highly correlated. It is important to include correlated brain regions in the same model, but under the traditional regression framework the simultaneous inclusion of highly correlated variables can cause a severe multicollinearity problem and lead to invalid statistical inference. A second issue is that brain regions interact with each other to fulfill a cognitive function. However, for a standard regression analysis, an exhaustive specification of all the interactions among brain regions is near impossible. A third consideration is that it may be overly simplified to assume that all brain regions relate to a cognitive function in a linear fashion. The random forest method is well equipped to handle these challenges. Random forest is an ensemble method that works by generating a large number of data sets via resampling with replacement from the original data set (bootstrap samples) and making a collective decision (e.g., association) by combining results from the analyses of all resampled data sets. Random forest has a built-in training and testing mechanism to overcome overfitting problems associated with traditional machine learning methods (Smialowski et al. 2010). Specifically, in each resampling procedure about two thirds of the original observations are included in the bootstrap sample, which is used to grow each tree in the forest. One third of the observations are left out to evaluate the predictive performance of the tree. The importance of each variable is assessed by randomly permuting the values of the variable in the sample that is left out of each resampled data set. If a variable is important in terms of its relationship with a measure, after the random permutation the performance using the permuted variable should decrease. Variables can therefore be rank ordered in terms of their importance.

### Intermediate analysis

To select sMRI predictor variables, an intermediate analysis was first conducted to identify regions that showed significant group differences in basal ganglia volume and cortical thickness. To account for the potential confounding nonlinear effect of age and the interaction between age and gender, random forest was used to control for the covariate effect of age and gender on brain morphometry in each region. Data from gene-negative controls were first used to derive the relationship of cortical thickness and basal ganglia volume with age and gender. The difference between observed and predicted thickness/volume was calculated from this fitting, which defined a set of residuals (residual 1). Then data from the prHD group were used to obtain the estimated effect of age and gender using the same model, and a second set of residuals were calculated (residual 2). Next, a two-sample Wilcoxon rank sum test compared residuals 1 and 2 for each cortical region and basal ganglia volume. Abnormal brain morphometry in prHD was declared if the mean residual 1 for a region was significantly greater than the mean residual 2. A false discovery rate (FDR) of 0.05 was used to adjust for multiple comparisons. Regions showing significant mean thinning or atrophy in the prHD group were then used as sMRI variables in the main statistical analyses.

### Main analyses

Random forest was used to model the relationship between the sMRI variables identified in the intermediate analyses and performances in each cognitive domain only in the prHD group. The analyses were conducted separately for each cognitive variable. To adjust for the confounding effects of age, gender, education, and number of visits on cognitive performance, these variables were also included in the random forest model. The number of bootstrap samples was set at 5000, and the default value of the number of predictors divided by 3 was used for the number of variables randomly sampled when assessing the importance of variables. The importance measure of each sMRI variable in relation to each cognitive measure was determined by the increase in mean squared error (MSE) in correlating with the outcome for observations outside the bootstrap sample when values of the sMRI variable were randomly permuted. The MSEs of all sMRI variables were ranked to quantify the relative importance of each brain region in correlating with the outcome of a cognitive measure. In order to obtain the most robust sMRI variable ranking, each random forest analysis was repeated 1000 times and the average ranking for each variable was used. To select the most parsimonious model that had at least as good performance as a model that used all sMRI predictors, the variable selection method of Genuer et al. (2010) was applied. By this method, the top ranking variables that rendered the smallest mean MSE over 200 runs in their correlation with performance on each cognitive measure were chosen for interpretation. Although random forest is a relatively complicated analytic method, it is surprisingly computationally efficient. For the analysis in our study, each random forest run took about 19 sec, although computation time depends on the hardware and operating system.

## Results

### Cognitive measures

**Figure 1:**
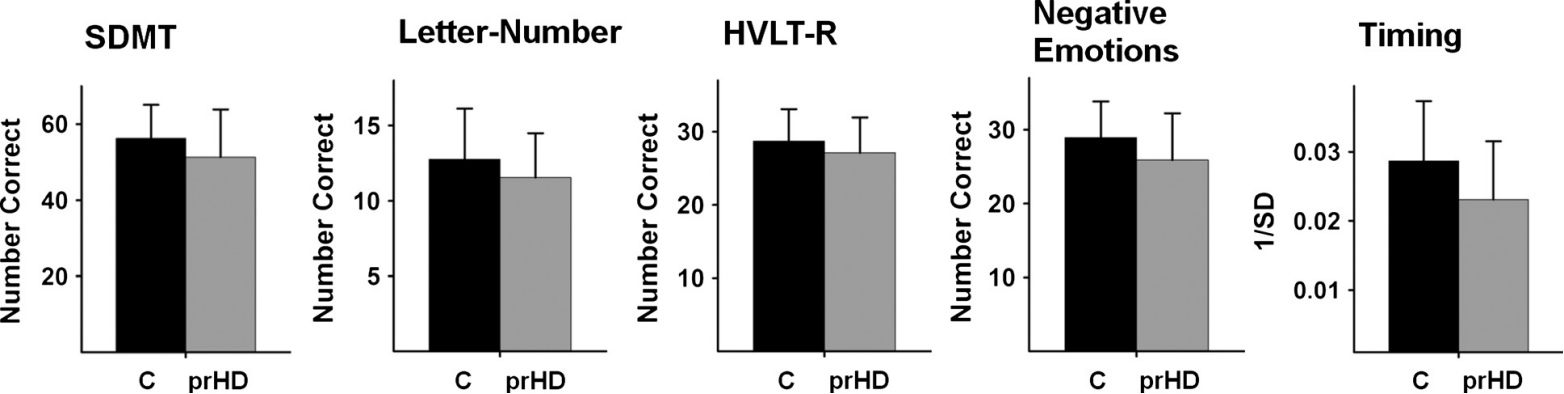
Mean (standard deviation) group performance on each of the cognitive measures. The gene-negative control group (C) performed significantly better than the prHD group on all cognitive measures (SDMT:*P* < 0.0025; letter-number sequencing: *P* < .013; HVLT-R:*P* < 0.037; negative emotions: *P* < 0.01; and timing: *P* < 0.002). prHD, prodromal Huntington disease; SDMT, Symbol Digits Modality Test; HVLT-R, Hopkins Verbal Learning Test-Revised.

To characterize the entire prHD group, an ANCOVA tested for group differences on each of the cognitive measures, adjusting for age, gender, years of education, and number of visits (*P* < 0.05, unadjusted). Figure 1 plots the means (standard deviations) for the groups on each measure. The prHD group performed significantly worse on all cognitive measures (SDMT: *t* = −3.04, *P* < 0.0025; letter-number sequencing: *t* = −2.50, *P* < 0.013; HVLT-R: *t* = −2.09, *P *< 0.037; negative emotions: *t* = −2.58, *P* < 0.01; and timing: *t* = −3.16, *P* < 0.002).

### Cortical thinning and basal ganglia atrophy in prHD

**Figure 2:**
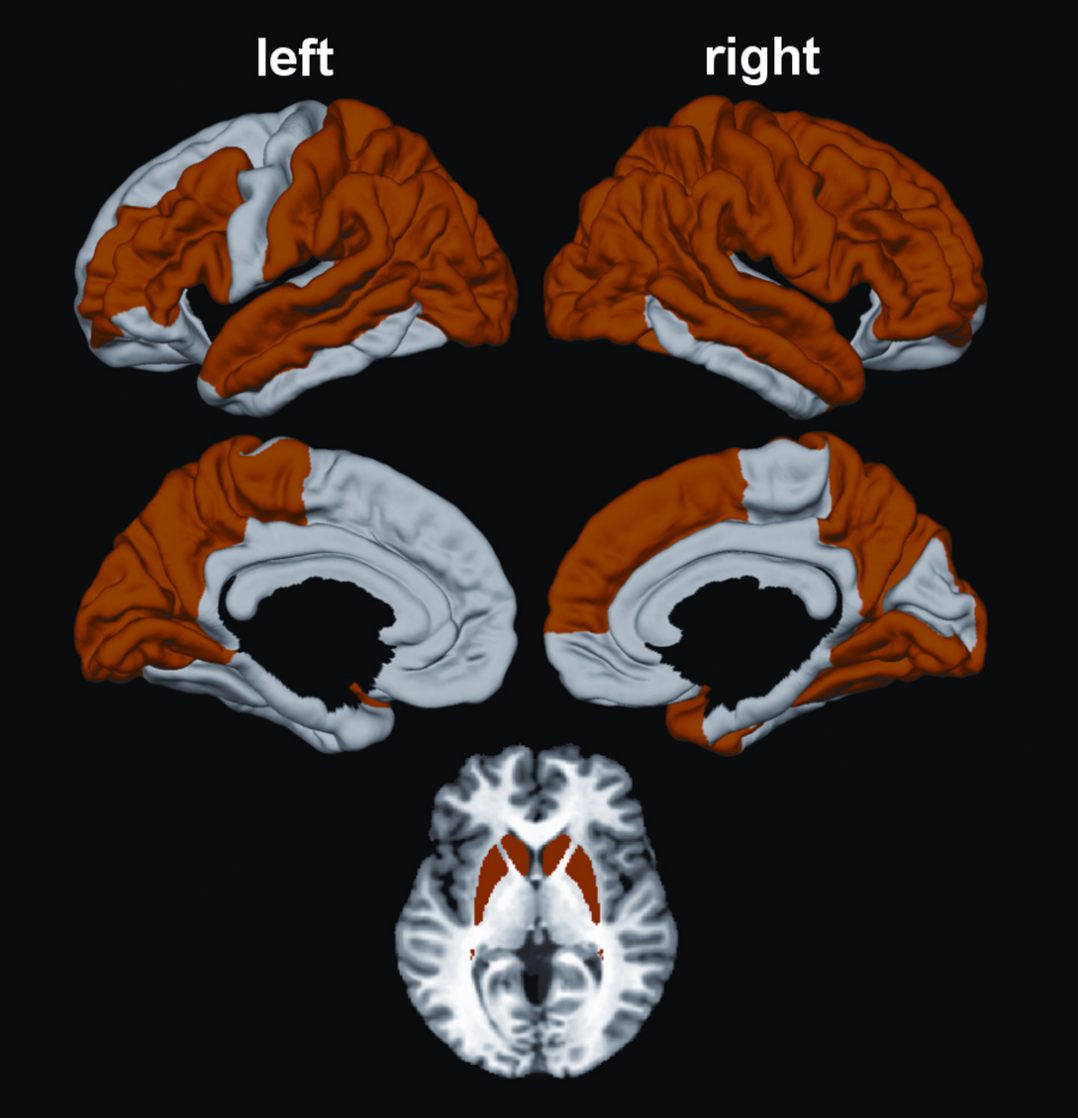
Regions showing significant cortical thinning and striatal atrophy in the prodromal Huntington disease (prHD) group. Bilateral caudate and putamen atrophy were found in the prHD group. Cortical thinning was also found in 36 regions including areas of the frontal, superior and middle-temporal, and parietal-occipital cortices of both hemispheres on lateral and medial surfaces. These 40 regions were the predictor variables in the random forest analyses.

Figure 2 displays regions showing significant mean basal ganglia volume loss and cortical thinning in the prHD group relative to the gene-negative controls. As expected, significant volume loss was found in the bilateral caudate and putamen. Cortical thinning was found in 36 regions including areas of the frontal, superior and middle-temporal, parietal, and occipital cortices of both hemispheres on the lateral and the medial surfaces. These 40 regions were used as sMRI predictors of performance in each cognitive domain.

### sMRI correlates of cognitive functioning

**Figure 3:**
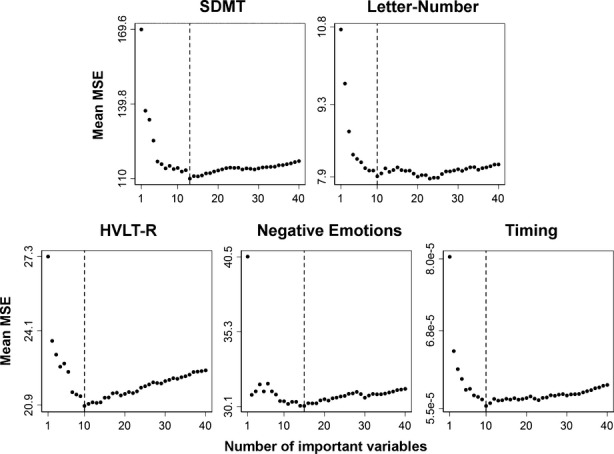
Number of top structural MRI (sMRI) correlates of performance for each cognitive measure. Each circle in the plot represents a sMRI predictor variable. The *x* axis shows the number of sMRI variables based on their mean squared error (MSE) ranking in the random forest analysis. The y axis represents the mean MSE value of the variables when the corresponding number of top sMRI predictors was included in the model. The lowest mean MSE is marked with a dashed line and signifies the number of top ranking variables that provided the most parsimonious correlation with performance on each cognitive measure. Negative emotions and SDMT performances were best associated with the highest ranked 15 and 13 sMRI variables, respectively. For the other cognitive variables, the 10 highest ranked sMRI variables resulted in the lowest mean MSE. An exception was for letter-number sequencing, in which the mean MSE was technically the lowest for the top-ranked 23 variables, but very close to the mean MSE corresponding to the top-ranked 10 sMRI variables. As such, the top 10 sMRI variables were selected for a more parsimonious interpretation.

**Figure 4:**
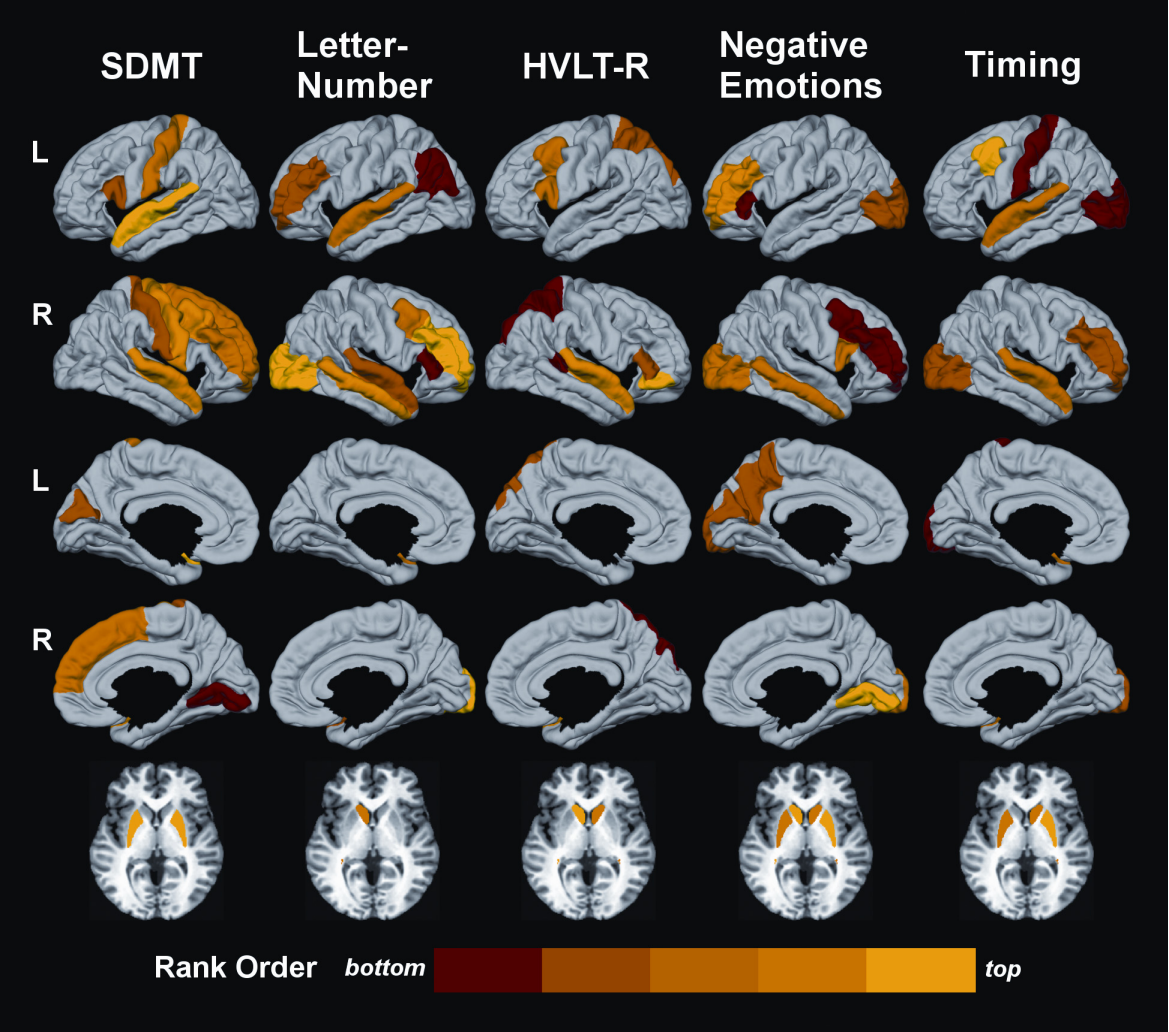
Spatial maps of the top-ranked structural MRI (sMRI) correlates of performance in each cognitive domain. Cortical regions are displayed on the lateral (1st and 2nd rows) and medial (3rd and 4th rows) surfaces of the left (L) and right (R) hemispheres. The basal ganglia are shown at the bottom. The importance of a brain region in correlating with a cognitive measure is color coded on a continuum (red to yellow) according to the rank order of the mean square error (MSE) value for a sMRI variable, where larger MSEs signified greater importance. Yellow signifies a higher rank order of importance than red. Colors on the bar designate variables ranked in the top 20th (yellow) to the bottom 20th (red) percentile of the top-ranked sMRI predictors for each cognitive measure.

Figure 3 shows the number of the top-ranked sMRI variables that minimized the mean MSE (designated by the dotted line) for each cognitive measure. Negative emotions and SDMT performances best correlated with the highest ranked 15 and 13 sMRI variables, respectively. For the remaining cognitive variables, the 10 highest ranked sMRI variables resulted in the lowest MSE. An exception was the letter-number sequencing task, wherein the MSE was technically the lowest for the top-ranked 23 variables, but very close to the MSE corresponding to the top-ranked 10 sMRI variables. With the principle of parsimony in mind, the model with the top 10 sMRI variables was selected for interpretation. Figure 3 also shows that as more sMRI variables were added to the model, there typically was a progressive reduction in the mean MSE until it was minimized. For the Negative Emotions task, however, the top two ranked variables almost minimized the mean MSE, although the addition of other sMRI variables did result in a slightly lower mean MSE. Figure 4 displays the spatial maps of the top-ranked sMRI correlates of performance for each cognitive measure according to their mean rank order of importance, with lighter colors corresponding to more highly ranked sMRI variables. The exact rank order of sMRI variable importance is listed in Table 2. An inspection of the data showed that for all top-ranked sMRI correlates of each cognitive measure, greater cortical thinning and striatal atrophy were associated with worse performance.

**Table 2:** Rank order of importance for the top sMRI correlates of performance in each cognitive domain

Rank order	SDMT	Letter number	HVLT-R	Negative emotions	Timing
1	R putamen	R occipital	L caudate	R putamen	L cMFG
2	L putamen	R rMFG	R pOrbt	R lingual gyrus	R putamen
3	L STG	L caudate	R STG	L caudate	L putamen
4	R STG	R MTG	R caudate	L putamen	R caudate
5	R precentral	L STG	L cMFG	R caudate	L STG
6	R cMFG	R cMFG	L pOper	L rMFG	R STG
7	R SFG	R STG	R pTrng	R pOper	R occipital
8	R rMFG	L rMFG	L SP	R occipital	R rMFG
9	L postcentral	L IP	R STS	R MTG	L occipital
10	L cuneus	R pTrng	R SP	L precuneus	L postcentral
11	R postcentral			L cuneus	
12	L pOper			L occipital	
13	R lingual gyrus			R rMFG	
14				L pTrng	
15				R cMFG	

L and R, left and right hemisphere; cMFG, caudal middle-frontal gyrus; IP, inferior parietal; MTG, middle-temporal gyrus; pOper, par opercularis; pOrbt, pars orbitalis; pTrng, pars triangularis; rMFG, rostral middle-frontal gyrus; SFG, superior frontal gyrus; SP, superior parietal; STG, superior temporal gyrus; STS, bank of the superior temporal sulcus; SDMT, Symbol Digits Modality Test; HVLT-R, Hopkins Verbal Learning Test-Revised.

Figure 4 shows that the top-ranked correlates of SDMT performance included elements of the motor circuit (bilateral putamen, right precentral gyrus, bilateral postcentral gyrus), right hemisphere cognitive-control centers in prefrontal cortex (PFC) (right superior frontal, caudal and rostral middle-frontal cortex), an auditory and semantic processing hub including Broca's area (left pars opercularis, bilateral superior temporal cortex), and visual centers (left cuneus, right lingual gyrus). The highest ranked sMRI variables were the bilateral putamen, followed by the bilateral superior temporal cortices and then right hemisphere PFC regions (Table 2).

Top-ranked correlates of letter-number sequencing performance included the striatal-frontoparietal working memory network (left caudate, bilateral rostral middle frontal, right caudal middle frontal, right pars triangularis, left inferior parietal), an auditory and semantic processing hub (left superior temporal), and elements of the right ventral attention network (right lateral occipital and middle-temporal cortices). The highest ranked sMRI variables were the right lateral occipital and right rostral middle-frontal cortices, followed by the left caudate and the right middle-temporal cortex (Table 2).

Top-ranked correlates of HVLT-R immediate recall performance included dorsal frontoparietal regions of the working memory network (bilateral caudate, left caudal middle frontal, bilateral superior parietal) and a semantic processing network including Broca's area (left pars opercularis, right pars triangularis and pars orbitalis, and right superior temporal gyrus and bank of the superior temporal sulcus). The highest ranked sMRI variables were the left caudate and right pars orbitalis, followed by the right superior temporal cortex and right caudate, and then other PFC regions (Table 2).

Top correlates of negative emotion performance included a frontostriatal cognitive-control network (bilateral caudate and putamen, bilateral rostral middle frontal, right caudal middle frontal, right BA pars opercularis, left pars triangularis), a memory retrieval hub (left precuneus), and visual processing regions (right lingual gyrus, bilateral lateral occipital cortex, left cuneus, and right middle-temporal cortex). The highest ranked sMRI variables were the right putamen and right lingual gyrus (Table 2).

Top correlates of motor timing precision included a frontostriatal cognitive-control network (bilateral putamen, right caudate, left caudal middle frontal, and right rostral middle frontal), sensorimotor cortex (left postcentral gyrus), and multimodal association centers (bilateral superior temporal and bilateral lateral occipital cortices). The highest ranked sMRI variable was the left caudal middle-frontal cortex, followed by bilateral putamen, right caudate, and bilateral superior temporal cortex (Table 2).

## Discussion

This study demonstrated that functioning in different cognitive domains that are vulnerable to decline in prHD is associated with regionally specific patterns of both cortical and striatal morphometry. Although caudate and/or putamen volumes in prHD are known to correlate with cognitive performances on several tests (e.g., SDMT, Stroop Interference, Verbal Fluency, WCST, Trail Making Test) (Campodonico et al. 1998; Jurgens et al. 2008; Paulsen et al. 2010; Wolf et al. 2013), most studies report no relationship between cortical volume loss or thinning and cognition (Novak et al. 2012; Wolf et al. 2013), with one notable exception (Rosas et al. 2005). This is surprising given the widespread changes in cortical morphometry in prHD (Nopoulos et al. 2010). Discrepant findings may relate to variations among studies in imaging processing methods, sample size, and levels of proximity to disease onset. Individuals far from diagnosis (more than 15 years) typically perform similarly to controls on most cognitive measures, whereas those closer to diagnosis perform more poorly relative to gene-negative controls (Stout et al. 2011). Likewise, striatal volumes decrease and cortical thinning increases with proximity to diagnosis (Nopoulos et al. 2010; Paulsen et al. 2010). However, individuals far from diagnosis do not exhibit significant cortical thinning (Nopoulos et al. 2010), although striatal volumes can be reduced. As such, the wide range of cognitive performances and cortical-striatal structure in this study provides a strong test of the anatomical correlates of cognitive functioning in prHD. Moreover, it is reasonable to infer that the structure–function relationships identified by our study are more expressed as disease burden advances. However, longitudinal studies are needed to directly evaluate this supposition. Our results build upon reports of cognitive-sMRI associations in combined samples of prHD and HD individuals (Bechtel et al. 2010; Say et al. 2011; Scahill et al. 2013) by elucidating sMRI correlates of cognitive functioning in different domains that are specific to the premanifest period.

One notable finding was that attention and information processing speed, as measured by the SDMT, was uniquely associated with thickness of both the motor (precentral gyrus) and sensory (postcentral gyrus) cortices and bilateral putamen volume. In fact, the bilateral putamen and right precentral gyrus were highly ranked correlates of performance. These results are compatible with the stronger sensorimotor component of the SDMT relative to most other cognitive measures except timing, which was also associated with sensory cortex thickness. The results also comport with the correlation of motor measures, such as maximum tapping speed (Bechtel et al. 2010) and visuomotor integration (Say et al. 2011), with sensorimotor cortex thinning in combined samples of prHD and HD participants, and the correlation of putamen, but not caudate volume, with SDMT performance in prHD (Jurgens et al. 2008). SDMT performance also depends on the capacity to selectively attend to and integrate symbol–digit pairs. This is consistent with its relationship to thickness in mostly right PFC executive-control centers and in an articulatory/semantic processing center (bilateral superior temporal cortex), which was also a highly ranked correlate of performance, perhaps because it assists in integrating symbol–digit pairs.

A distinctly different regional pattern of sMRI variables was associated with letter-number sequencing, which emphasizes executive components of working memory (i.e., manipulation of information) more so than the other tests. Performance was associated with thinning in elements of an executive working memory network, including the inferior parietal cortex and bilateral rostral PFC, which is thought to be engaged by more abstract or complex executive processes than caudal PFC (Badre 2008). Unlike the other cognitive domains, the highest ranked cortical correlates of performance were the right rostral middle-frontal cortex and the right lateral occipital and middle-temporal cortices, which by way of interactions with the PFC, selectively enhance the processing and maintenance of information in working memory (Lee and D'Esposito 2012). The greater importance of rostral PFC in its association with working memory is consistent with functional magnetic resonance imaging (fMRI) studies of verbal working memory, which report hypoactivation and weakened connectivity of the dorsolateral FC (DLPFC) in prHD (Wolf et al. 2007, 2008). Although working memory was not related to cortical thinning in another study (Wolf et al. 2013), this result may be due to the small sample size (*n* = 20) and/or the use of different working memory tasks (spatial and digit span), which may not emphasize executive aspects of working memory to the same extent. Another top-ranked correlate of working memory ability was the left caudate, consistent with its anatomical connections with the rostral PFC, especially the DLPFC.

Cortical thickness in a decidedly more dorsal frontoparietal working memory network was associated with verbal learning ability on the HVLT-R, including bilateral superior parietal cortex and the caudal PFC, which presumably modulates less abstract executive-control processes (Badre 2008). However, the left caudate was the highest ranked variable of performance, perhaps because the striatum governs updating and integrative functions of working memory (Hazy et al. 2007), which is vital for learning. Other top-ranked variables were components of the articulatory and semantic processing network including Broca's area (superior temporal and inferior frontal cortices), consistent with the emphasis of the HVLT-R on verbal rehearsal.

The ability to recognize negative emotions was associated with yet another regional pattern of corticostriatal morphometry in structures commonly associated with emotion processing including the bilateral caudate and putamen, a memory encoding/retrieval center (precuneus), and visual analysis centers of the occipitotemporal cortices (lingual gyrus, cuneus, lateral occipital cortex, and middle-temporal cortex) (Adolphs 2002). These results are compatible with an fMRI study reporting temporal-occipital hypoactivation in prHD during an *implicit* emotion processing task (Novak et al. 2012). However, the same study found no relationship between cortical morphometry and *explicit* negative emotion recognition in prHD (Novak et al. 2012), possibly due to the small sample size (*n* = 16) and normal task performance. An important consideration is that in our study the two top-ranked correlates of negative emotion recognition, namely, right putamen and right lingual gyrus, minimized most of the MSE suggesting that the morphometry of these structures in prHD was most highly associated with task performance. Putamen volume, especially the ventral portion, and lingual gyrus thickness may be critical because these structures, respectively, modulate limbic system processing and govern refined visual analyses, which is especially important for recognition of negative facial expressions. Although orbitofrontal cortex is more commonly associated with emotion processing, this region was not included in our analyses as there was no significant atrophy in the prHD group. The amygdala also mediate negative emotion recognition (Adolphs et al. 1999), but amygdala volumes were not available, which is a limitation of this analysis.

Lastly, we found that motor timing precision was also associated with the caudate and putamen, PFC cognitive-control centers, and temporal-occipital regions. The highest ranked variables were the left caudal middle-frontal cortex, followed by the putamen/caudate, and then bilateral superior temporal cortex. These findings comport with striatal modulation of a core timekeeping system, which is thought to receive and integrate duration information about relevant events from the PFC and multimodal association regions (Harrington et al. 2010; Merchant et al. 2013). Our results are compatible with an fMRI study reporting hypoactivation of the striatum during motor timing in prHD (Zimbelman et al. 2007). This study also reported hyperactivation of the bilateral superior temporal cortex in individuals who were more than a decade from diagnosis, but not in individuals closer to a manifest diagnosis. It is unknown whether hyperactivation reflects compensation, but our results suggest the possibility that individuals with more significant atrophy may not be capable of compensation because performance is impaired. Whether presumed compensatory responses are related to the structural integrity of brain tissue is an important area for future investigations.

## Conclusions

This study uncovered distinct regional patterns of cortical and striatal morphometry that correlated with functioning in different cognitive domains in the prHD group. Although the volume of one or more striatal nuclei was typically one of the higher ranked correlates of functioning across domains, cortical thickness of various brain regions was also a top-ranked correlate of all cognitive functions. It is unlikely that co-occurring psychiatric symptoms in prHD were a factor in our results, as gray matter volume was unrelated to psychiatric measures in a large combined sample of prHD and early diagnosed HD patients (Scahill et al. 2013). Furthermore, co-occurring depressive symptoms in prHD do not correlate with proximity to diagnosis (Epping et al. 2013), unlike motor and cognitive symptoms and gray matter volume and thinning. Certainly, functional imaging studies are needed to better illuminate neurocognitive relationships, but our results suggest the possibility that the functionality of brain circuits may partly depend on their structural integrity. Structural changes may not affect functioning unless there is sizeable atrophy or thinning, although longitudinal studies of sMRI-cognitive correlates are needed to confirm and extend these findings. Another important consideration is that white matter volume and tissue diffusivity changes in prHD also influence cognitive functioning (Magnotta et al. 2009; Paulsen et al. 2010; Aylward et al. 2011; Dumas et al. 2012; Matsui et al. 2013) via weakening of corticostriatal and corticocortical communication. Thus, multimodal imaging approaches, including diffusion tensor imaging, will likely be the future path toward delineating the earliest changes in the brain, elucidating their functional significance, and tracking the timescale of progression.

As for clinical applications, our study highlights the importance of identifying sMRI markers of functioning in different cognitive domains, as their relative sensitivity depends on the extent to which processing is called upon by different brain networks. This information will inform clinical trials where there is a need to use cognitive and neuroimaging outcomes that are relevant to the treatment target(s). Moreover, the search for a single “best” neural marker of cognitive decline is likely to be misguided, as behavior depends on complex interactions among brain regions. With the application of more powerful statistical methods such as random forest, one can begin to utilize knowledge about the importance of multiple predictors, which exhibit complex relationships with behavior, to guide the selection of clinical outcome measures. This feature of random forest, together with its more generalizable and robust results relative to single sample analysis (Berk 2006), may further prove to be more sensitive in identifying combinations of neurobiological markers that are sensitive to the earliest changes in prHD, wherein treatment effects are more likely to succeed.
